# Oral Resveratrol supplementation attenuates exercise-induced Interleukin-6 but not Oxidative Stress after a high intensity cycling challenge in adults

**DOI:** 10.7150/ijms.55633

**Published:** 2021-03-18

**Authors:** Jung-Piao Tsao, Chia-Chen Liu, Hsueh-Fang Wang, Jeffrey R. Bernard, Chun-Ching Huang, I-Shiung Cheng

**Affiliations:** 1 Department of Physical Education, National Taichung University of Education, Taichung City, Taiwan.; 2 Department of Nutrition, Institute of Biomedical Nutrition, Hungkuang University, Taichung City, Taiwan.; 3 Department of Kinesiology, California State University, Stanislaus, Turlock, CA, USA.; 4 Department of Exercise and Health Science, National Taipei University of Nursing and Health Science, Taipei City, Taiwan.

**Keywords:** antioxidant phytochemicals, ergogenic property, cycling exercise, fatigue

## Abstract

Previous studies demonstrated that resveratrol (RES) is able to enhance antioxidant, anti-inflammatory and insulin actions in humans. It is unclear whether RES can be used as ergogenic aids to enhance high-intensity cycling exercise performance and attenuate the high-intensity exercise-induced oxidative stress and inflammation. This study investigated the effect of RES supplementation on oxidative stress, inflammation, exercise-induced fatigue, and endurance performance. Eight male athletes participated in this single-blind crossover designed study and randomly instructed to receive four days of either oral RES (480 mg per day, totally 1920mg) or placebo supplementation. The cycling exercise challenge at 80% maximal oxygen consumption with 60 rpm was performed following 4 days of either RES or placebo supplementation. The total cycling performance time was recorded. In addition, blood samples were obtained to analyze the changes in blood glucose, plasma non-esterified fatty acid, serum lactate dehydrogenase, creatine kinase, uric acid, total antioxidant capacity, malondialdehyde, tumor necrosis factor-α, and interleukin-6. The exhausting time of cycling exercise challenge was not significantly increased in RES compared to that in placebo. However, IL-6 response was significantly decreased during exercise challenge in RES trial, and there were no differences in blood biomarkers, fatigue factors, and antioxidative response. Oral RES supplementation can attenuate exercise-induced IL-6 response but not fatigue and oxidative stress, inflammation response. However, we infer that 4-day oral RES supplementation has no ergogenic property on enhancing the high-intensity cycling exercise performance.

## Introduction

Resveratrol (trans-3, 4', 5-trihydroxystilbene, RES) is a naturally occurring polyphenol 1 with antioxidant 2, anti-inflammatory 3 properties. Resveratrol reported to increase insulin sensitivity 4 and accelerates fat oxidation 5. However, the effect of RES supplementation on oxidative stress, inflammation and energy metabolism remains unclear in humans following exercise challenge. High-intensity exercise trigger reactive oxygen species (ROS) production and cause oxidative stress and inflammation in humans, which eventually aggravate fatigue and decrease exercise performance 6, 7. Pro-inflammatory cytokines, including interleukin-6 (IL-6) and tumor necrosis factor-alpha (TNF-α) were reported to increase in rat skeletal muscle after a 90-min downhill running 8. Administration of RES (50 mg/kg) with self-nanoemulsifying drug delivery system exerted anti-fatigue effect in exhaustive swimming rats 9. Another study demonstrated that rats with low doses of RES for 21 consecutive days significantly enhanced the swimming endurance performance. The supplementary effect of RES was notable regardless of its dosage (25, 50 or 125 mg/kg), and a dose-dependent effect was observed due to its antioxidant and anti-inflammatory properties 10.

Human trials revealed that RES supplementation improved maximum oxygen uptake (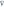
O_2max_), Wingate anaerobic test peak power, peroxisome proliferator- activated receptor gamma coactivator 1-α (PGC-1α), Sirtuin 1 (SIRT1) level and superoxide dismutase 2 (SOD2) activity after 4-week high-intensity interval training. However, the synergistic effects of RES supplementation and exercise training were not observed according to this human study by Scribbans 11. Another human study demonstrated that shorter-term RES supplementation (4-day) could not enhance glycogen replenishment and mitochondria biosynthesis in exercised skeletal muscle following a 70% 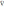
O_2max_ cycling exercise challenge for 60 minutes 12. The physiological impact of high-intensity exercise may have masked the RES effect on augmenting the performance in these two human studies.

Interleukin-6, a pro-inflammatory cytokine is an important indicator of glycogen metabolism during exercise period 13, which concomitants a progressive decline of muscle glycogen content after a prolong exercise in humans 14. High-intensity cycling exercise produced an abruptly systemic IL-6 response involving a significant muscle mass in the contractile activity. Of course, oxidative stress induced after a high-intensity exercise challenge can increase pro-inflammatory cytokines, such as IL-6 and tumor necrosis factor-α (TNF-α) reactions 15, 16. Therefore, attenuation of pro-inflammatory response may depend on the rapid elimination of oxidative stress during athletic performance. *In vitro* or animal studies showed that RES significantly reduces oxidative stress and positively affects anti-inflammatory reactions 17. In particular, animal studies have shown that RES significantly alleviates exercise-induced fatigue and suppressed oxidative stress 10,18,19. It has been further documented that the oral bioavailability of RES was increased and the swimming time to exhaustion was also significantly increased after RES using self-micro emulsifying drug delivery system 9.

4-day oral RES supplementation (480 mg per day, totally 1920 mg) was conducted in the present human study. The dosage of resveratrol used and duration of supplementation period employed from the calculating on the efficient rodent dosage and duration 9. The relevant human study of similar resveratrol products was conducted by Huang et al. on muscle cell mitochondria biosynthesis with 4 days of oral supplementation during the post-exercise recovery period 12. Therefore, the purpose of our human study was to clarify the short-term ergogenic property of resveratrol on attenuating oxidative stress, pro-inflammation, as to eliminate exercise-induced fatigue under the cycling exercise at 80% 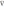
O_2max_ with 60 rpm.

## Materials and Methods

### Participants

Eight male participants, physically active students, with an average age of 19.2 ± 0.5 years and an average body mass index (BMI) of 23.3 ± 1.8 and an average 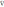
O_2max_ of 51.4 ± 1.7 mL/kg/min were recruited. All participants attended the experiment design explanation and provided informed consent before beginning the experimental process. During the experimental period, the participants were required to avoid consuming pungent beverages (e.g., coffee, tea, cola, and chocolate), drugs and nutritional products which caused oxygen stress, inflammation response or changes in metabolism status will be suggested to refrain from consuming by subjects during experimental period. The investigations were carried out following the rules of the Declaration of Helsinki of 1975 and this study was approved on August 1st, 2017 by the Institutional Review Board in University of Taipei, Taipei, Taiwan (approval license IRB-2016-053). All participants joined the experiment voluntarily, for 2 months prior to the formal experiment and avoided taking supplements with anti-inflammatory agents. All subjects were fully informed of the risks and discomfort associated with the study, and all provided voluntary written consent before participation.

### Experiment design and procedure

All subjects performed this single-blind crossover study design with a 7-day washout period between each trial. The participants' maximal oxygen consumption (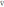
O_2max_) test was measured 7 days before each single bout of exercise challenge. Starting 3 days before each exercise challenge, the participants were reminded to avoid intense exercise performing, coffee consumption, and smoking. The participants consumed 300-calorie breakfasts each day (60% carbohydrates, 25% fat, and 15% protein), and received 480-mg RES supplements or placeboes; on the fourth day, 60 min after RES supplementation, the participants began to perform a cycling challenge (Monark Exercise, Varberg, Sweden). A 5-min warm-up exercise was conducted with a resistance of 50 W. Then, a 60-rpm cycling endurance test was performed at 80% of 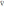
O_2max_, and the time of challenge completion was recorded. The participants were asked to stop the workout and record the time after meeting two of three conditions: (1) unable to maintain the default exercise intensity with heart rate at maximum (220 - age); (2) oxygen uptake no longer increasing and respiratory exchange ratio (RER) > 1.1; (3) rated perceived exertion (RPE) level ≥ 18. Before, during, and after each workout, blood samples were collected to measure the concentrations of glucose, non-esterified fatty acids (NEFA), lactate dehydrogenase (LDH), creatine kinase (CK), uric acid (UA), total antioxidant capacity (TAC), malondialdehyde (MDA), TNF-α, and IL-6.

### Maximal oxygen consumption test

The participants wore masks to complete the 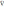
O_2max_ measurement on cycle ergometers while using gas analyzers. The ergometers were required to maintain a rotating speed of 60 rpm. The load was initially set at 0.5 kg for 4 min, and then increased by 0.5 kg at 2-min intervals until the participants were exhausted. The 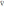
O_2max_ was required to satisfy the following criteria: (1) RER > 1.10; (2) 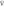
O_2max_ variance < 2 mL/kg/min; and (3) heart rate target reaches maximum heart rate at (220-age) 20.

### RES and placebo supplementation

During the 4-day RES supplementation, the participants consumed breakfast in the laboratory at 8:00 AM each day. After breakfast, the participants received oral RES supplementation comprising three 160-mg capsules (a total of 480 mg) or three placebo capsules. The RES capsule contained a mixture of resveratrol with maltodextrin and lecithin (Taiwan Jellyfig Biotechnology Corporation, Kaohsiung, Taiwan), the placebo capsules with starch. The RES capsules were nanocapsules created using self-microemulsifying drug delivery system technology, containing 160 mg of RES extracted from peanuts. These RES capsules had higher absorption rate than that of commercially available RES 9.

### Blood biochemical assessments

Vacuum blood collection tubes containing heparin anticoagulant were employed to collect blood samples from the radial veins of the participants. The samples were centrifuged at 4 °C and 1,000 *g* for 10 min. Plasma from the supernatant was then collected and preserved at -80 °C in a freezer.

Blood glucose concentration was determined by an automated glucose analyzer (YSI Life Sciences). NEFA, LDH, CK, and UA concentrations were measured using the commercialized assay kit with an automatic photometric analyzer (Hitachi 7020, Japan), NEFA reagents (Randox, Ransel, UK), and LDH, CK, and UA reagents (Kanto Chemical, Kanagawa, Japan). The results were converted to concentration units. TAC and MDA indices, which are relevant to antioxidant capacity and oxidative stress respectively, were measured using a commercial TAC and MDA reagent (Cayman Chemical Company, ANN Arbor, MI, USA). An enzyme immunoassay (Tecan GENios, A-5082, Austria) was applied to measure the absorbance at 570 nm and the TAC and MDA concentrations in the serum samples were calculated according to the standard curve. The concentrations of TNF-α and IL-6, which are cytokines, were measured using commercially available IL-6 (R & D, HS 600C, Minneapolis, USA) and TNF-α (R & D, HSTA00E, USA) reagents, respectively. An enzyme immunoassay (Tecan GENios, A-5082, Austria) was applied to measure the absorbances of IL-6 and TNF-α at 540 and 405 nm, respectively; the concentrations of IL-6 and TNF-α in the serum samples were then calculated according to the standard curves.

### Statistical analysis

All data were represented as mean with their standard errors (Mean ± SE) using SPSS software (IBM SPSS Statistics; IBM Corp.). A paired *t*-test was conducted to examine the differences between the participants in the performance of cycling challenge. Two-way ANOVA with repeated measures was used to compare all blood samples. If a significant interaction was detected between the experimental trials and the time, a simple main effect analysis was conducted, and Fisher's post hoc test was used to distinguish significant difference between pairs of conditions. The α level was set at 0.05 to indicate a significant difference for all comparisons.

## Results

### Effects of Resveratrol supplementation on cycling time to exhaustion and energy substrates metabolism

**Figure 2:**
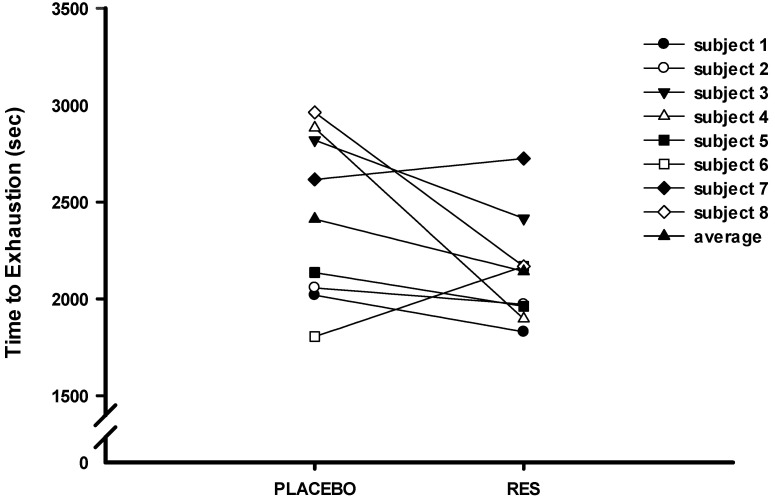
Individuals/average time to exhaustion (TTE) with 80% 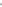
O_2max_ exercise challenge after placebo or resveratrol supplementation.

**Figure 3:**
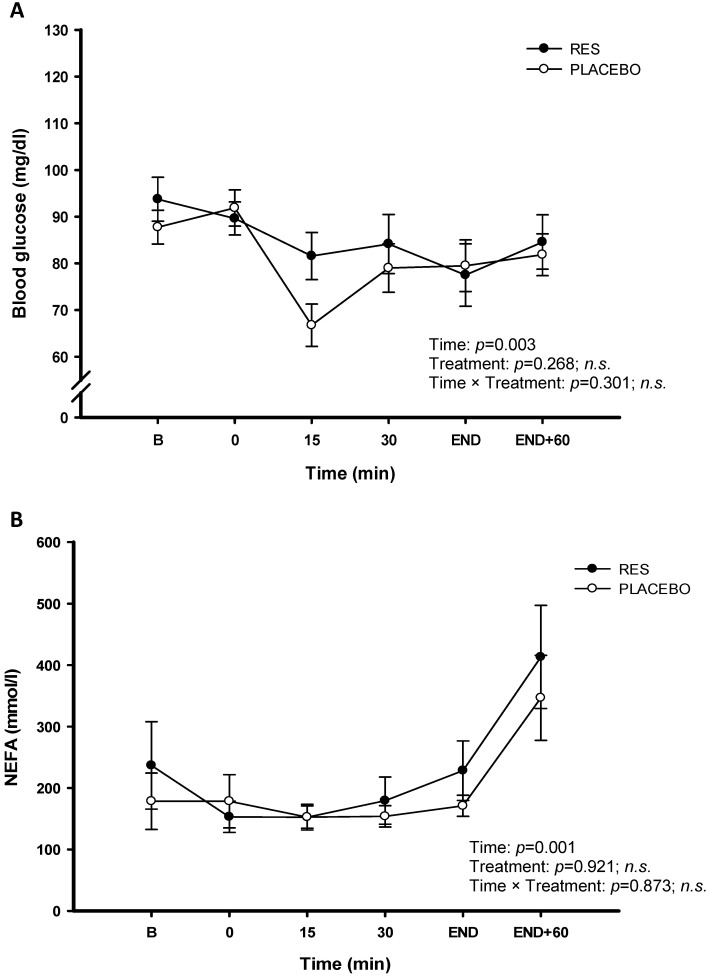
Blood glucose (A) and plasma non-esterified fatty acids (NEFA) (B) concentrations before and during exercise in resveratrol (-●-) and placebo (-○-). B represents before exercise. END represents immediately finishing exercise. END+60 represents 60 minutes after exercise. Values are expressed as mean ± SE, N=8. * Significant difference against placebo (*P* < 0.05).

The results of this study on the individuals' or statistically average exhausting time under a single bout of high-intensity cycling exercise challenge were not significantly increased after oral RES supplementation (RES: 2142.25 ± 106.48 (sec); Placebo: 2412.5 ± 161.41 (sec), *p* > 0.05) (Figure 2). Identically, the blood glucose (Figure 3A) and NEFA (Figure 3B) did not significantly different response after oral RES supplementation compared to those of placebo treatment (*p* > 0.05).

### Resveratrol supplementation attenuates IL-6 but not oxidative stress

**Figure 4:**
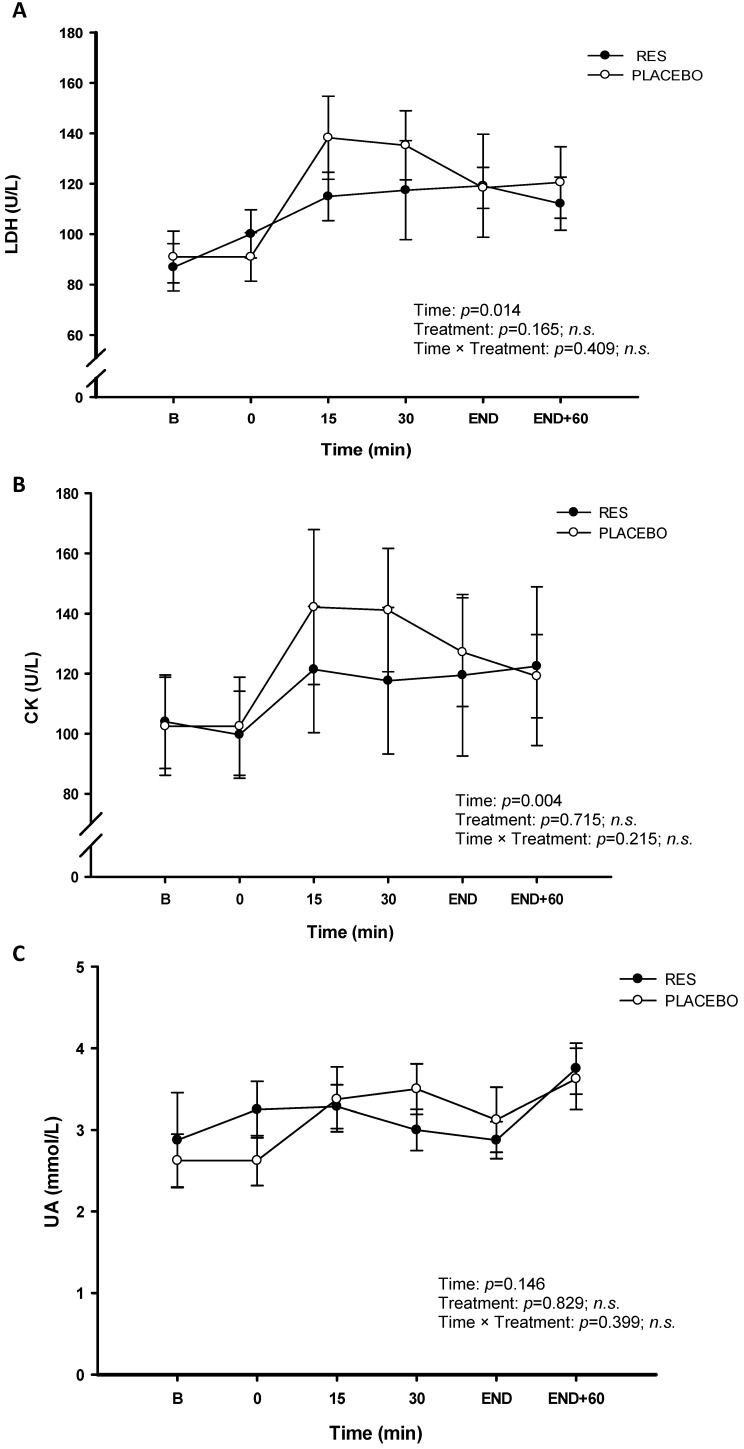
Serum lactate dehydrogenase (LDH) (A), creatine kinase (CK) (B) and uric acid (UA) (C) concentrations before and during exercise in resveratrol (-●-) and placebo (-○-). B represents before exercise. END represents immediately finishing exercise. END+60 represents 60 minutes after exercise. Values are expressed as mean ± SE, N=8. * Significant difference against placebo (*P* < 0.05).

**Figure 5:**
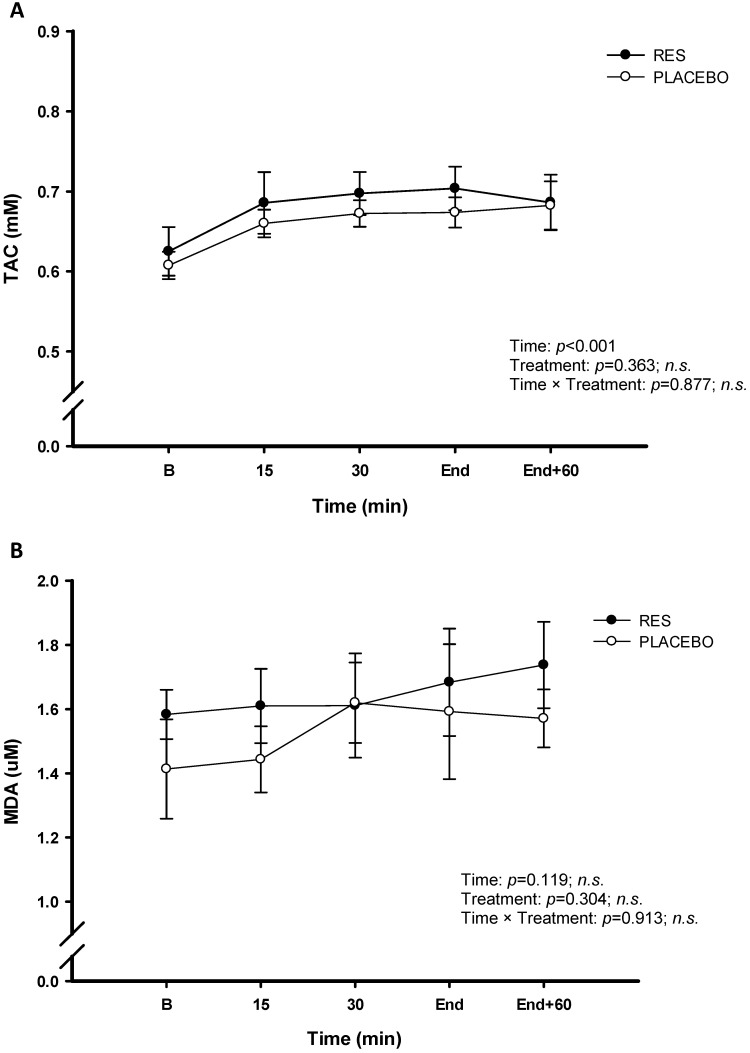
Serum total antioxidant capacity (TAC) (A) and malondialdehyde (MDA) (B) concentrations before and during exercise in resveratrol (-●-) and placebo (-○-). B represents before exercise. END represents immediately finishing exercise. END+60 represents 60 minutes after exercise. Values are expressed as mean ± SE, N=8. * Significant difference against placebo (*P* < 0.05).

**Figure 6:**
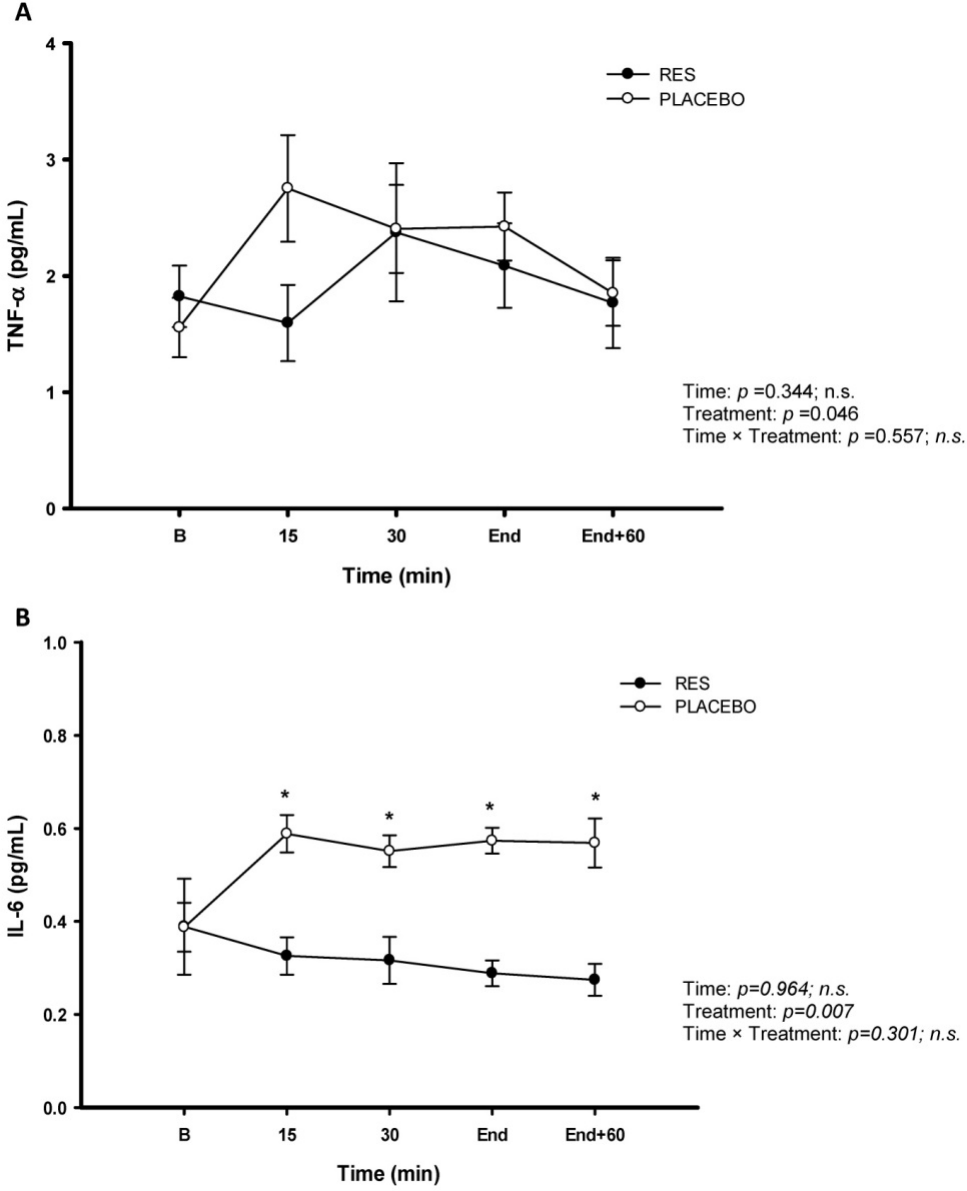
Serum tumor necrosis factor-α (TNF-α) (A) and interleukin-6 (IL-6) (B) concentrations before and during exercise in resveratrol (-●-) and placebo (-○-). B represents before exercise. END represents immediately finishing exercise. END+60 represents 60 minutes after exercise. Values are expressed as mean ± SE, N=8. * Significant difference against placebo (*P* < 0.05).

The concentrations of the LDH (Figure 4A), CK (Figure 4B), and UA (Figure 4C) were no significant difference between RES and placebo (*p* > 0.05). The cycling exercise performance were not enhanced after 4-d RES supplementation, based on no difference in energy substrates metabolism and attenuating tendency in exercise induced fatigue between two trials. The changes in inflammation and oxidative stress were measured during this high-intensity cycling exercise challenge. The figure 5 showed oxidative stress indicators TAC (Figure 5A) and MDA (Figure 5B). Exercise induced oxidative stress indicators TAC and MDA were shown that there were no significant differences between two trials (*p* > 0.05). Figure 6 showed no significant differences pro-inflammation indicators of TNF-α between two treatments (Figure 6A, *p* > 0.05). However, there was significantly lower exercise-induced response in IL-6 after oral resveratrol supplementation (Figure 6B, *p* < 0.05).

## Discussion

In the present study, RES supplementation before the high-intensity exercise was assumed to enhance anti-oxidation and anti-inflammation capabilities, mitigate factors contributing to exercise-induced fatigue, and improve participants' cyclic exercise performance. After 4 days of receiving oral RES supplementation, the participants engaged in a high-intensity cycling challenge. The results were as follows: (1) the single bout of exercise performance with 80% 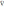
O_2max_ at 60 rpm did not be improved during the cycling challenge; (2) the blood parameters of glucose, NEFA, LDH, CK, and UA measured during exercise did not support the hypothesis that RES attenuates exercise fatigue; (3) the plasma TAC, MDA and TNF concentrations indicated that RES supplementation did not improve the antioxidant and anti-inflammation capabilities. (4) RES supplementation significantly decreased the IL-6 level induced by exercise stimulus. According to cell experiments, RES supplementation increased the number of muscle mitochondria in endothelial cells 21. Low doses of RES supplementation significantly increased muscle endurance and power in swimming animals 10. Cell and animal experiments have indicated that RES supplementation improves antioxidant 3 and anti-inflammation 22 capabilities in animals. In the present human experiment, RES supplementation did not effectively improve high-intensity cycling exercise performance or mitigate the exercise-induced fatigue. However, RES supplementation seems to prevent muscle stress on lower IL-6 release, a cytokine with pro-inflammation, during the high-intensity cycling exercise challenge following 4-day oral RES supplementation.

IL-6 is a pro-inflammatory cytokine and muscle damage marker following exercise challenge 13, which has positive association with content of contracting skeletal muscle glycogen 23. The rats study showed that a single bout of a 90-min downhill running eccentric exercise significantly increased mRNA IL-6 and TNF-α but no change in serum levels of IL-6 and TNF-α 8. The human study showed that 65% and 85% maximal power (W_max_) of knee extension exercise were performed for 35 min separately in seven healthy males and the authors inferred that IL‐6 may be linked to the regulation of glucose homeostasis during exercise 24. In our human study, the significantly lower IL-6 response during the exercise period was found after RES ingestion, we implied that 4-day RES supplementation prevented the release of cytokine with pro-inflammation during high-intensity cycling exercise. Although there was no measurement of muscle glycogen in the present study to support this positive effect of RES. However, IL-6 is an important metabolic role during exercise that mediates the effect of lipolysis, inflammation, and muscle glycogen metabolism 25. Therefore, we infer that the significantly lower IL-6 during exercise in this study may be implied the 4-day RES supplementation have ergogenic property on preventing of muscle cytokine with pro-inflammation or attenuating on exercising muscle glycogen depletion during high intensity cycling exercise.

The individual observation of this study revealed that only two of the participants' have shown the improved cyclic exercise performance after the 4-day RES supplementation, and two of the remaining participants have very poor performance than those of placebo supplementation. This was possibly because the initial intensity of the bicycle ergometer overwhelmed the leg muscles of the participants, who expressed severe fatigue in their legs. Exercise fatigue is caused by the overconsumption of energy in human bodies during high-intensity exercise, which produces excessive free radicals and leads to high oxidation and inflammation in the body. During exercise, the glycogen in skeletal muscles is rapidly exhausted, causing rapid blood glucose consumption and accelerating lipolysis. Under this condition, NEFA concentration, which is a blood biochemical index for lipolysis, increases to provide the energy needed to maintain the exercise performance 26. CK and LDH in the blood are a well-established marker of muscle damage used to infer damage to the sarcolemma. The significant changes of CK and LDH were observed when the load of a workout exceeding the body's capacity, which triggering the exercise-induced fatigue to attenuating on the high-intensity exercise performance 27. High blood urea also indicates that more protein is broken down than is synthesized during exercise fatigue 28. However, the blood glucose, NEFA, CK, UA, and LDH concentrations measured in the present study could not confirmed the mitigating effect of RES supplementation on high-intensity exercise fatigue. There was no treatment effect of RES on the blood indices in the present human study. Although the application of nanoemulsifying-RES capsules eliminated problems related to RES absorbance and bioavailability 9, 10, 29, RES supplementation did not mitigate the factors related to exercise fatigue or improve the exercise performance of the participants in the cycling challenge.

During high-intensity exercise periods, ammonia, a protein metabolite, is broken down into urea. Plasma levels of urea are known to increase in response to exercise. During fatiguing exercise, active skeletal muscle oxidation of purines increases, thus increasing the efflux of uric acid from skeletal muscle into the blood compartment 30, As uric acid is an antioxidant this should increase TAC 31. In present human study, the data of TAC showed an increase over time during exercise period after RES supplementation (Figure 5A). Additionally, creatine kinase is a marker for a muscle tissue which may be correlated with physical training status, as depend on sarcomeric damage 32. The highest post-exercise serum enzyme activities are found after prolonged exercise such as ultradistance marathon running or weight-bearing exercises 33, 34. LDH catalyzes the conversion of pyruvates to lactic acids and is an index of anaerobic metabolism during high-intensity exercise 35. However, after 4-day RES supplementation, high LDH, UA, and CK concentrations were identified in the placebo groups during the cycling challenge. Although the LDH, UA, and CK concentrations in the RES group were not as high as those in the placebo group, no statistical difference was identified between the two groups. The results of our study implied the positive effect of 4-day RES supplementation could not be supported on attenuating exercise fatigue in exercising humans. We speculated the dosage and supplement duration as the crucial factors in our human study from the previous resveratrol effect on human interventional trials 36. The relevant human study revealed that 4 days RES (1920 mg totally) failed in enhancing muscle glycogen recovery and mitochondrial biosynthesis 12. Therefore, the total dosage is insufficient on influencing the changes in exercising metabolites due to duration of the supplementation in the present study. Further human studies will be conducted using the recommended dosage of 60 mg/d for chronic effect and 2400 mg/d for acute effect of resveratrol 9, 37, which are warranted to confirm the ergogenic properties on endurance performance.

RES supplementation is likely to exert anti-fatigue pharmacological effect in animal studies 9, 10. In the present human study, we speculated that the same trend may occur in TAC, MDA, and TNF-α after RES supplementation. However, the 4-day RES supplementation in the present study did not show the positive effect under high-intensity cycling performing based on TAC, MDA and TNF-α. This was inconsistent with the findings of numerous cell and animal experiments, which have indicated that RES supplementation regulates antioxidant and anti-inflammatory functions 38, 39. However, exercise-induced IL-6 exists lower response in this study. Therefore, we conclude that 4-day oral RES supplementation can attenuate exercise-induced IL-6 response but not fatigue, oxidative stress. We inference that 4-day oral RES supplementation have positive influence on cytokine IL-6 but no ergogenic property on the high-intensity cycling exercise performance. However, this study also has limitations including the recruitment number of participates although a single-blind crossover study design was conducted. Further future studies need to attend to sample size. Similarly, there was a lack of a diet diary and records about physical activity for all participants. Based on the knowing variation of MDA as a measure of oxidative stress, diet and training diaries could provide detailed information that could clarify the shorter-term ergogenic property of resveratrol on oxygen stress, inflammation status and high-intensity cycling performance-enhancing in humans.

## Conclusions

In summary, we demonstrate that 4-day oral RES supplementation (480 mg/day) is capable of attenuating the IL-6, cytokine with pro-inflammatory, during high-intensity cycling exercise. However, the given RES dosage and supplement duration appear to be insufficient to attenuate oxygen stress and fatigue during exercise. The final outcome demonstrated the 4-day oral RES supplementation (480 mg/day) could serve as ergogenic aid on attenuating IL-6 but not oxygen stress and fatigue during high-intensity cycling exercise. Since inflammation is an important factor for endurance performance, this finding could certainly beneficial to athletes, who are training for key competition.

## Figures and Tables

**Figure 1:**
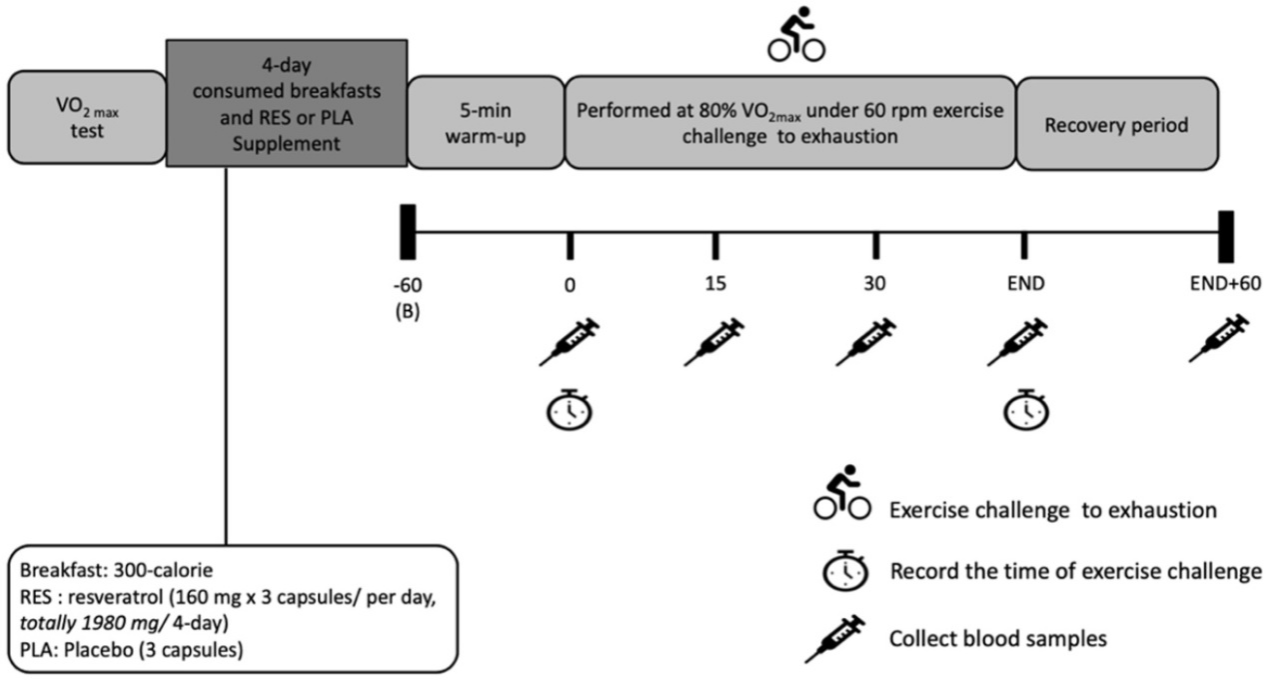
Experimental design and protocol.

## Abbreviations

RES: resveratrol
NEFA: non-estrified fatty acid
LDH: lactate dehydrogenase
CK: creatine kinase
UA: uric acid
TAC: total antioxidant capacity
MAD: malondialdehyde
TNF-α: tumor necrosis factor-α
IL-6: interleukin-6
RER: respiratory exchange ratio
RPE: rated perceived exertion
